# Cytotoxicity and Synergistic Effect of Biogenically Synthesized Ternary Therapeutic Nano Conjugates Comprising Plant Active Principle, Silver and Anticancer Drug on MDA-MB-453 Breast Cancer Cell Line

**DOI:** 10.31557/APJCP.2020.21.1.195

**Published:** 2020

**Authors:** Arjunan Karuppaiah, Ravikumar Rajan, Muthiah Ramanathan, Arumugam Nagarajan

**Affiliations:** 1 *Department of Pharmaceutics, *; 2 *Department of Pharmacology, *; 3 *Department of Pharmacognosy, PSG College of Pharmacy, Coimbatore, Tamil Nadu, India. *

**Keywords:** Breast cancer, silver nanoparticles, doxorubicin, andrographis paniculata, synergistic effect

## Abstract

Drug delivery through biogenically synthesized silver nanoparticles (AgNPs) in cancer treatment is exerted by smaller size entailing high surface area and synergistic effects of embedded biomolecules. In this study, prepared ternary conjugates of silver with plant active compound and anticancer drug towards reducing the dose through synergy, rendered by Electrostatic Attraction (EA) of functionalized drug on to the surface of biogenically synthesized AgNPs. The biogenic synthesis resulted in particles of nanometer range as well as serving reducing and capping agents. The cytotoxicity and synergistic effect of ternary therapeutic nano conjugates evaluated using MDA-MB-453 breast cancer cells were found to be superior than Doxorubicin (Dox). Quantitative HPTLC analysis showed 57.22 % inhibition by Dox-AP-AgNPs at a concentration of 2.5 µg/mL of *Andrographolide* and 0.95 µg/mL of Dox validating synergistic effect of the ternary conjugate.

## Introduction

Breast cancer accounts for nearly a quarter (23%) of all cancers in women and diagnosis at developed stages causes higher mortality rates necessitating technological breakthroughs in terms of easy availability, cost effectiveness and safety of therapeutic intervention (National Cancer Registry Programme., 2013). Biogenically synthesized nanosilver based drug delivery in cancer treatment has significant potential due to its smaller size, high surface area and synergistic effects of embedded systems. Doxorubicin (Dox) owes its anti cancer activity to specific DNA intercalation affecting macromolecular biosynthesis but suffers cardiotoxicity. A review on the utility of Dox loaded nanoparticles in breast cancer therapy appeared in 2012 (Prados et al, 2012). 

Nanotechnology provides nano-sized particles with high surface area that manifest improved physicochemical properties over their bulk materials (Li et al., 2011). Silver nanoparticles (AgNPs) have a vital function against AIDS virus and particularly cancer (Nirmala et al., 2012, Shin et al., 2007, Ma et al., 2011, Lu et al., 2008). AgNPs are significantly more toxic than Ag^+ ^to prokaryotic cells and have been shown to be effective bactericides at nanomolar concentrations compared with µM levels for Ag^+ ^(Choi et al., 2008). Synthesis of AgNPs through physical and chemical processes have been replaced by green synthesis that has proved to be an easier and alternative method mainly because of employing microorganisms (Ahmad et al., 2003), plant extracts (Daphedar et al., 2017) and milk (Lee et al., 2013. 

In this work we proposed a novel synthetic scheme to produce a AgNPs core shell surrounded Dox conjugate by Electrostatic Attraction (EA) to be used as an active agent against breast cancer cell line, MDA-MB-453. Green chemistry process envisaged for the preparation of ternary therapeutic nano conjugates (Plant active principle, Silver and Anticancer drug) to provide maximum cytotoxicity and synergistic effects on MDA-MB-453 human breast cancer cell Line. The plant active compounds chosen is *Andrographis paniculata (AP)* because of its role in suppressing cancer progression (Venkatadri et al., 2016, Babykutty et al., 2008, Kamdem et al., 2002). *Andrographis paniculata (AP)* (Ahmad et al., 2014) has found extensive use in traditional medicines for anticancer, nutritional benefits (Flamini et al., 2013, Esfahanian et al., 2013) and other activities. 

## Materials and methods

*Materials*

Doxorubicin HCl was provided by RPG Life Science Ltd (Fermentation division), Gujarat, India. Silver nitrate was obtained from Sigma-Aldrich Chemicals. Analytical quality chemicals and reagents were used in this study. Milli-Q Elix and Simplicity 185 purification system was employed to get Ultra pure water used in the experiments. 

*Selection of plant materials*

The plant chosen for the biogenic synthesis of silver nanoparticles was authenticatedat Botanical survey of India, Southern regional centre, Coimbatore, *Andrographis paniculata*: BSI/SRC/5/23/2015/Tech./2294. The leaf parts of *AP* were procured from local vendors of Coimbatore, Tamil Nadu, India. Washing with tap water and finally with double-distilled water ensured that the removal of dirt and other foreign materials.

*Preparation of the plant extracts*

*AP* leaves were collected and shade dried. 100 gm of powdered leaves were extracted with 200 mL of methanol using Soxhlet extractor for 12–24 hours. The extract was concentrated in rotary vacuum evaporator and stored in amber glass bottles until use. 

*Synthesis of silver nanoparticles*

Biogenic synthesis of AgNPs has been carried out using the *AP* plant extract. The active components present in the extract served as reducing as well as capping agent. The AgNPs were prepared by taking 10 mL of 1.0 mM silver nitrate solution in a beaker to which 1 mL of plant extract was added drop by drop, maintaining the reaction mixture under vigorous stirring using a magnetic stirrer for 10 min at room temperature. Initially the solution turned to light yellow and then to yellowish brown color. 

*Preparation of ternary nanocomposite*

In a typical procedure, to 5 mL of AgNPs solution taken in a beaker, 1 mL of 1 mg/mL Dox aqueous drug solution was added drop by drop and magnetically stirred at room temperature for 18 hours. The free silver ions (Ag^+^), plant metobolites and Dox from the ternary NPs was purified by ultra centrifugation method. Doxorubicin-silver nanoparticles (Dox-*AP*-AgNPs) were centrifuged at 13,000 rpm for 45 min to ensure the adsorption of Dox on *AP*-AgNPs till saturation and remove the free Dox, Ag^+ ^and plant extract metabolites (Wang et al., 2012).

*Characterization of nanoparticles*

Preliminary characterization of the *AP*-AgNPs was carried out using UV-spectroscopy using a Shimadzu dual-beam spectrophotometer (model UV-1601 PC). The average mean diameters and Poly Dispersive Index (PDI) of nanoparticles was found by photon correlation spectroscopy using Zetasizer (nano ZS90, Malvern Instruments) at 25^o^C. The zeta potential (ZP) measurements of nanoparticles were made using Zetasizer (Nano ZS90, Malvern Instruments). FT-IR spectra were recorded using Thermo Nicolet, Avatar 370, and spectral range of 4,000 to 400 cm^-1^ Transmission Electron Microscopic (TEM) analysis was done using TEM, JEOL JEM-2100 instrument operated at an accelerating voltage at 80 kV. The Energy Dispersive Analysis of X-ray (EDAX) observations were carried out in JEOL JEM-2100. The Selected Area Electron Diffraction (SAED) patterns were carried out in JEOL JEM-2100 and the nanocrystallites were analyzed using Quanta 200 FEG. The pH value measurements of the *AP*-AgNPs were carried out in Systronics µ pH system 361. In order to check the stability of the synthesized *AP*-AgNPs, they were stored in well closed containers and kept at 4.0±2.0^o^C and 23.0±2.0^o^C, respectively. Stability assessments of nanoparticle were characterized periodically for their average particle size, zeta potential analyses, PDI, and plasmon resonance absorbance.

*High performance thin layer chromatography (HPTLC) quantification*

Quantification of DOX and *Andrographolide* was done by HPTLC method. Standards and Nanoparticles samples were chromatographed with pre coated TLC plates. Standard *Andrographolide*, *AP* extract, *AP*-AgNPs and Dox-*AP*-AgNPs were developed in chloroform : toluene : methanol (6 : 2.6 : 0.8, v/v/v). Dox and Dox-*AP*-AgNPs were developed in chloroform : methanol : acetic acid : water (8 : 2 : 1.4 : 0.6, v/v/v/v).

*Antioxidant Assay*

Antioxidant activity of biogenically reduced *AP*-AgNPs, Dox-*AP*-AgNPs, crude extract (5 mg/mL in water), and Dox (5 mg/mL in water) were determined using DPPH (0.2 mM in methanol) free radical scavenging assay and ascorbic acid (1.0 mM in methanol) was used as reference standard for comparison.

*2,2-diphenyl-1-picrylhydrazyl (DPPH) free radical scavenging assay*

DPPH assay was performed in a 96 wells plate using single concentration (20 mcL) method. An aliquot of 20 mcL of test sample (Dox, *AP*-AgNPs, Dox- *AP*-AgNPs, *AP* extract and Ascorbic acid was poured in to respective well of plate followed by the addition of 180 mcL of DPPH solution to each well making 200 mcL final volume. The mixture was incubated at room temperature for 30 min. Color changes from violet to yellow was observed due to the antioxidant property of the components. Absorbance of reaction mixture was measured on microplate reader at 517 nm. The antioxidant scavenging activity of formulations was calculated by using given equation.

% Scavenging Activity = (Abs of control– Abs of test sample /Abs of control)×100

Where, Abs- Absorbance

*3-(4,5-Dimethylthiazol-2-yl)-2,5-diphenyltetrazolium bromide (MTT) Assay*

To determine the cytotoxic effect of the prepared nanoformulations the conventional MTT assay was done. Briefly, MDA-MB-453 cells were purchased from NCCS, Pune, India and were seeded in 96 well plates at the density of 3,000 cells/well and incubated for 24 hours in humidified incubator maintained at 37°C and 5% CO_2_. Cells were seeded in 200 mcL of Dulbecco’s Modified Eagle’s Medium supplemented with 10% FBS solution. After 24 hours when cells are attached, the existing media was removed and replaced by fresh media along with various concentrations of the prepared AgNPs viz., 1, 3, 5, 10, 30, and 100 mcg/mL and incubated for 24 hours. After an incubation period of 24 hours, 20 µL of MTT solution (5 mg/mL) prepared in sterile PBS pH 7.4 was added and kept for 4 hours incubation. After 4 hours of incubation, the MTT solution was discarded and 100 mcL of DMSO solvent was added in each well and the optical density of the formazan product was read at 570 nm in a microplate reader. Percentage cell inhibition was calculated using following formula:

100- (At-Ab/Ac-Ab)×100

Where, 

At, Absorbance of test drug

Ab, Absorbance of blank

Ac, Absorbance of control

*Statistical Analyses of Data*

Statistical analyses were performed using Prism software (Version 5, GraphPad, GraphPad Software Inc., La Jolla, CA, USA). Results are expressed as the mean of three replicates ± standard deviation (SD). A level of statistical significance between groups was analyzed by One & two-way ANOVA. P<0.05 were considered statistically significant.

## Results

**Figure 1:**
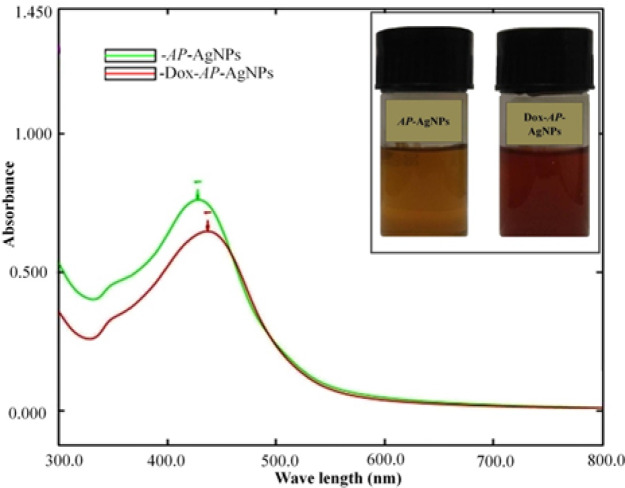
Plasmon Resonance Peak of *AP*-AgNPs and Dox-*AP*-AgNPs, Inlet Image is Formulations of Biogenic Silver Nanoparticles

Biogenic synthesis of metallic nanoparticles is a fast, clean and eco-friendly alternative and we have reported a simple and efficient method for the synthesis of AgNPs from silver nitrate solution using *AP* extract. Plant crude extract, a rich source of functional organic molecules has the ability to reduce Ag^+ ^in AgNO_3_ solution. The color change from colorless to yellow due to the formation of plasmonic resonance light scattering of AgNPs, indicates the formation of AgNPs in the mixture. The color of the solution gradually intensified with incubation time and turned into dark yellowish brown after few hours as shown in Figure 1. Drug adsorption onto metallic surfaces is determined by the intrinsic positive or negative charge possessed by both drug and metallic nanoparticles. The AgNPs which is having electro negative charge is electrostatically conjugated with the drug Dox having electro positive charge as reported by Sooresh A et al for the electrostatically conjugated nano silver to the pyrethroid pesticide deltamethrin (Sooresh et al., 2011). Dox resistance has been overcome by several techniques. Prominent among them are the use of Dox in combination with resveratrol nanoparticles (Fornari et al., 1994, Zhao et al., 2016), paclitaxel loaded lipid nanoparticles (Dong et al., 2009), intracellular drug delivery of Dox tethered responsive gold nanoparticles (Wang et al., 2011).

*Characterizations of nanoparticles*

The formation of AgNPs was confirmed by monitoring the mixture using a UV–Vis spectrometer at 300 to 800 nm. The maximum absorbance of formulations *AP*-AgNPs and Dox-*AP*-AgNPs, were noticed at 417±0.577 and 437±1.00 nm respectively (p< 0.0001) (Table 1), we found slight plasmon peak shift to longer wave length after drug conjugation in the formulation Dox-*AP*-AgNPs. The plasmon peak shift to longer wave length confirms the drug conjugation with *AP*-AgNPs, when the particle size increases the plasmon peak shift to longer wavelength. Usually AgNPs exhibits a strong and broad absorption peak close to 390 nm as a surface plasmon resonance, which confirms that Ag^+ ^were reduced to Ag° in the aqueous phase (Zielińska et al., 2009). We found formulation *AP*-AgNPs to have maximum absorbance at 417±0.577 nm presumably due to the color intensity of the *AP* reduced AgNPs, thus same *AP* reduced AgNPs with Dox given peak at 437±1.00 nm after few hours, the color of the solution gradually intensified with incubation time. The absorbance at longer wavelengths for the formulation indicates the formation of silver nanoparticle through drug adsorption onto the metal surface. It has been reported that the plant secondary metabolites such as polyphenols, triterpene saponin, stilbene derivatives, anthocyanins, alkaloids carboxylic acid, gallic acid, tannins, anthocyanins, and other components and flavonoids, present in the plant extract might be responsible for the reduction of Ag^+ ^to AgNPs resulting in alteration of the wavelength of absorption (Mittal et al., 2014, Shaik et al., 2018). Flavonoid compounds present in the extracts appear to be responsible for the formation of AgNPs by facilitating the reduction of Ag^+ ^to silver nuclei (Ago). 

The pH of *AP*-AgNPs was initially measured and followed by the drug Dox was adsorbed with *AP*-AgNPs and again the pH of Dox-*AP*-AgNPs was measured. The *AP*-AgNPs showed slight color changes from slight yellow to dark yellowish brown and notably formulation *AP*-AgNPs, but did not show any aggregations. Also analytical evaluations revealed that formulation *AP*-AgNPs and Dox-*AP*-AgNPs are stable to an optimum level. Here the *AP*-AgNPs which is reduced biologically has electro negative charge and the drug Dox has electro positive charge. The drug is electrostatically adsorbed on the surface of *AP*-AgNPs due to opposite charges. The pH values for *AP*-AgNPs was 7.3±0.152. The values for Dox-*AP*-AgNPs was 4.3±0.52 (p< 0.0001) (Table 1). The EA forces decreased at higher pH value as the size of the ion atmosphere around the polyions increased with increased salt concentration. The pH of *AP*-AgNPs was reduced after the EA of Dox, which indicates clearly the strong EA between anionic metallic nanoparticles and cationic drug particles, in conformity to be observation that at low pH values a long range of EA is present (Sooresh et al., 2011).

**Figure 2.**
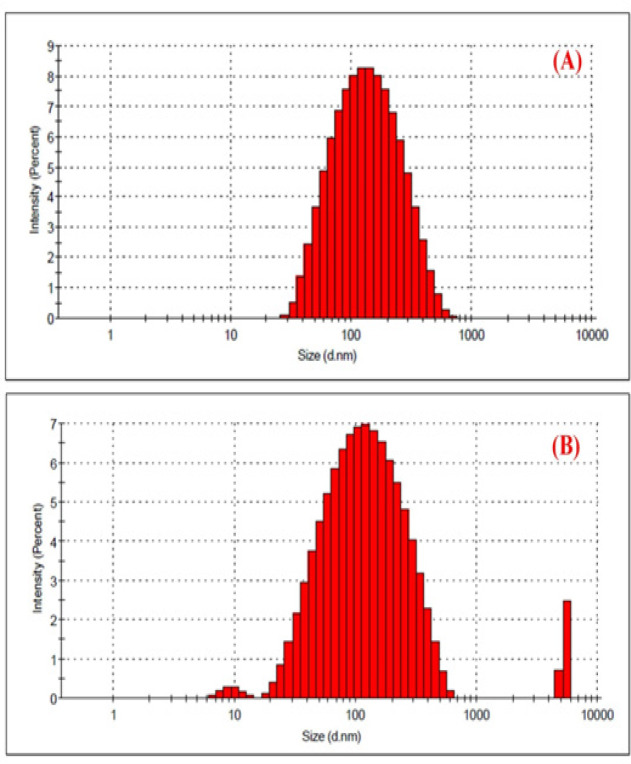
Particle Size Distribution. (A), *AP*-AgNPs; (B), Dox-*AP*-AgNPs

The average mean diameter of obtained biogenic *AP*-AgNPs was found to be 100±0.577 nm. The value for Dox-*AP*-AgNPs was found to be at 119±4.16 nm (p< 0.0001). The slight increase in particle size of the nanoparticles after the conjugation of drug indicates the arrangement of drug particles onto the metallic surface (Table 1). The PDI of the prepared nanoparticles were 0.586±0.0 and 0.637±0.0 respectively, for *AP*-AgNPs and Dox-*AP*-AgNPs which indicates the particles were well reduced and distributed evenly and there were no aggregation. The particle size distribution graphs show the mono dispersibility of *AP*-AgNPs and Dox-*AP*-AgNPs, Figure 2A shows that *AP*-AgNPs does not have any multiple peaks but Figure 2B shows Dox-*AP*-AgNPs have one another peak around 1000 nm, it may be due to the unadsorbed free Dox. The unadsorbed free Dox was removed by ultra centrifugation. 

The increase in zetapotential values in Dox-*AP*-AgNPs indicates drug particles are conjugated with AgNPs. Further, the electrokinetic properties of the *AP*-AgNPs were found to be -32.26±0.472 mV. The value for Dox-*AP*-AgNPs was -19.8±0.264 mV (p< 0.0001). We found zeta potential changes in the formulation *AP*-AgNPs after drug conjugation. Typically all the plant mediated AgNPs has potentially negative charge, and the drug Dox posses potentially positive charge, the arrangement of positively charged Dox to the surface of negatively charged AgNPs may reduce the zetapotential of AgNPs due to its surface adsorption. The increase in zeta potential of the ternary conjugate compared to the *AP*-AgNPs confirms the surface modification in the conjugate.

**Figure 3:**
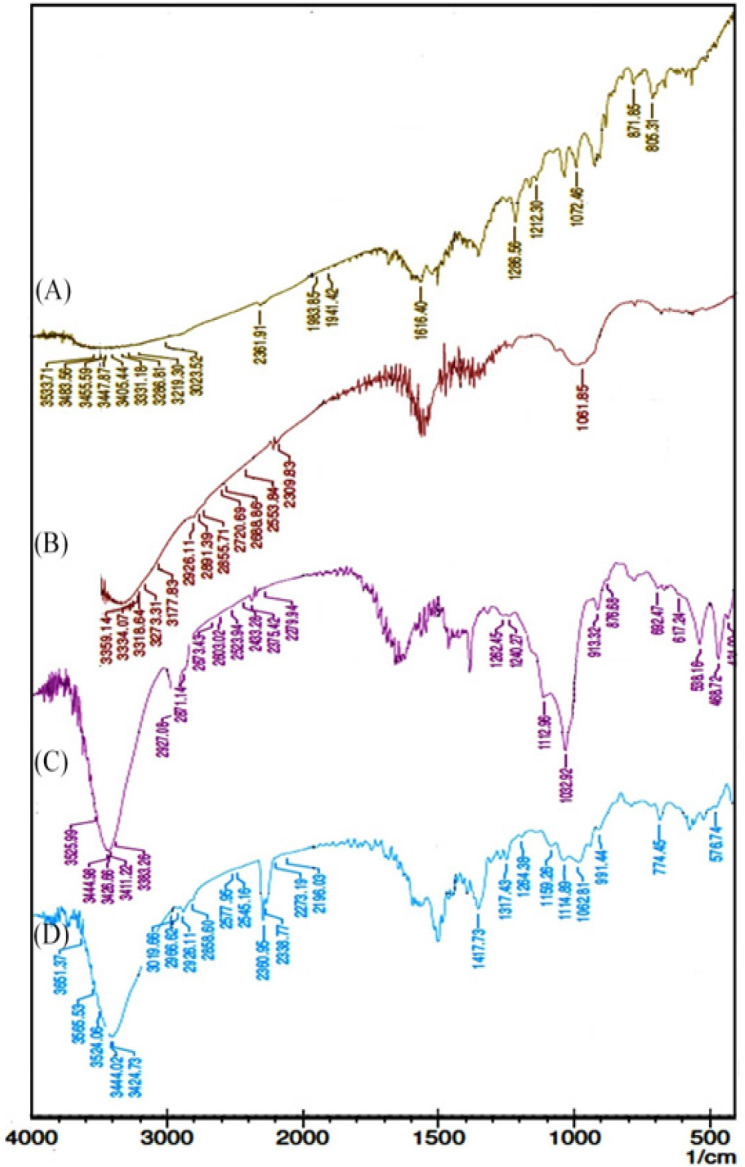
FT-IR Spectrum. (A), Dox; (B), *AP*; (C), *AP*-AgNPs; (D), Dox-*AP*-AgNPs

FT-IR spectrum was analyzed to identify the compatibility, conjugation and possible biomolecules responsible for the reduction of the Ag^+ ^ions in addition to surrounding the *AP*-AgNPs formed through the biogenic method. The characteristic peaks of Dox (Figure 3A) are 3410, 1638, 1338, 1065 (Quinone and ketone carbonyl), 871 (N-H wagging) and 761 cm-1 (N–H deformation) (Yuan et al., 2010). The bands of *AP* extract (Figure 3B) observed at 2926.11 cm-1 have been assigned to asymmetric –CH stretch of –CH3. The symmetric –CH was noted at 2,855.71 cm^-1^, free OH attached to the lactone ring demonstrated a peak at 3,383.26 cm^-1^, C=C of the diterpene ring demonstrated a peak at 1,450 cm^-1^, O-C=O in the lactone ring demonstrated a peak at 1357 cm^-1^ (Shivali et al., 2012). On the other hand, FT-IR spectrum of synthesized *AP*-AgNPs (Figure 3C) shows the major peaks at, 1,656, 2,375.42, 2,927.08, 3,383.26 and 3,444.98 cm^−1^ out of which 1,656, 2,375.42 and 3,444.98 cm^-1^ correspond to that of *AP*-AgNPs (Vivek et al., 2011). 

FT-IR spectrum of Dox-*AP*-AgNPs (Figure 3D) shows the major peaks at 3,424.73, 1,616, 2,926.11, 3,383.26, 2,360.95, 1,656 and 3,444.02 cm^−1^ and the peaks at 2,360.95, 1,656 and 3,444.02 cm^−1^ correspond to Dox-*AP*-AgNPs. In Dox-*AP*-AgNPs formulation we found a FT-IR signature peak of Dox around 3,400 cm^−1^ which strongly confirms the adsorption of drug with biogenic *AP*-AgNPs. Absence of peaks at 1,506 and 1,550 cm^−1^ correspond to nitrate group indicate that the AgNO_3 _has been converted to Ago. Further absence of peaks corresponding to 1,728 cm^−1^ for lactone ring suggests that the lactone ring might have opened up to form complex with Ago (Stephens et al., 2012). This further indicates that the lactone, after ring opening is not present as COOH (absence of 1,700 cm^-1^). The confirmation for the embedding of plant constituent and Dox on *AP*-AgNPs is confirmed by the retention of signature peaks in the ternary conjugate. 

**Figure 4:**
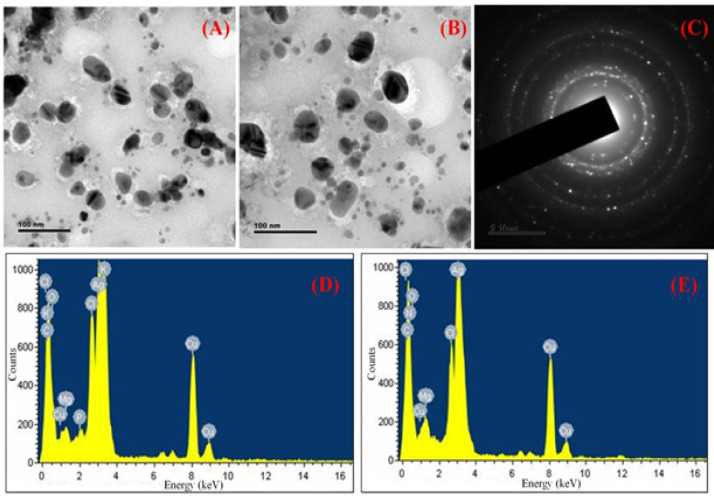
(A), TEM images of formulation *AP*-AgNPs; (B), TEM images of formulation *AP*-AgNPs after conjugated with Dox; (C), Scattered electron diffraction pattern of *AP*-AgNPs; (D), EDAX Spectrum of *AP*-AgNPs; (E), EDAX Spectrum of Dox-*AP*-AgNPs

TEM micrographs of the biogenically reduced *AP*-AgNPs showed less than 50 nm mono dispersed spherical nanoparticles (Figure 4A). The size of the observed particles, below 50 nm is in agreement with the average particle size values obtained by DLS. The TEM results are indicating the formation of *AP*-AgNPs through the biogenic synthesis and we observed the particles size of *AP*-AgNPs increase after adsorption with Dox. The Dox-*AP*-AgNPs also showed spherical shape with mono dispersed particles. There was no aggregation found in Dox-*AP*-AgNPs after drug conjugation (Figure 4B). The nano size of the ternary complex suggests that there could be a favorable synergistic effect of these novel combinations because of their smaller size and surface properties. 

Typical SAED patterns are, simple spot corresponding to single-crystal diffraction and ring patterns corresponding to multiple crystals of variable orientation. The SAED diffraction pattern obtained from *AP*-AgNPs were simple spot corresponding to single-crystal diffraction randomly deposited on the TEM grid (Figure 4C). The *AP*-AgNPs denotes particles were of single nano crystalline form in the formulation, as there is a simple spot corresponding to single-crystal diffraction.

EDAX gives qualitative status of elements that may be involved in the formation of *AP*-AgNPs. The EDAX of *AP*-AgNPs formulations shows only silver signature peaks (Figure 4D), whereas the EDAX of Dox-*AP*-AgNPs drug conjugate shows signature peaks for both silver and Dox (C_27_ H_30_ Cl N O_11_) functional elements such as N, O and Cl (Figure 4E). The EDAX data shows the presence of Ag, N, O and Cl in the formulation Dox-*AP*-AgNPs confirming the conjugation of Dox on to *AP*-AgNPs.

*HPTLC quantification*

**Figure 5:**
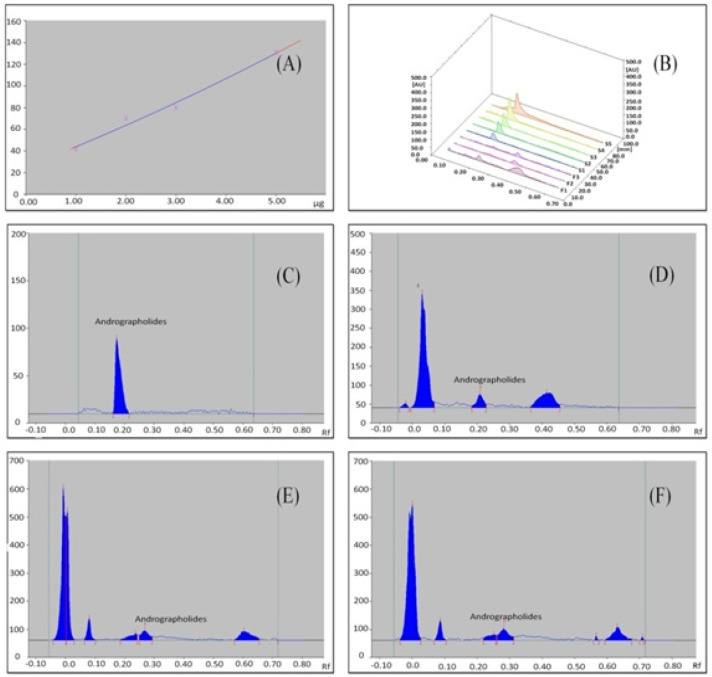
(A), Calibration curve of standard Andrographolide; (B), Densitometric chromatogram of Andrographolide at 254 nm (3D View), S1–S5 concentrations 1, 2, 3, 4, 5 mcg/spot standard Andrographolide, F1-*AP* extract, F2-(*AP*-AgNPs), F3-(Dox-*AP*-AgNPs); (C), Densitometric chromatogram of standard Andrographolide after derivatization at 254 nm; (D), Densitometric chromatogram of AP extract after derivatization at 254 nm; (E), Densitometric chromatogram of *AP*-AgNPs after derivatization at 254 nm; (F), Densitometric chromatogram of Dox-*AP*-AgNPs after derivatization at 254 nm

**Figure 6:**
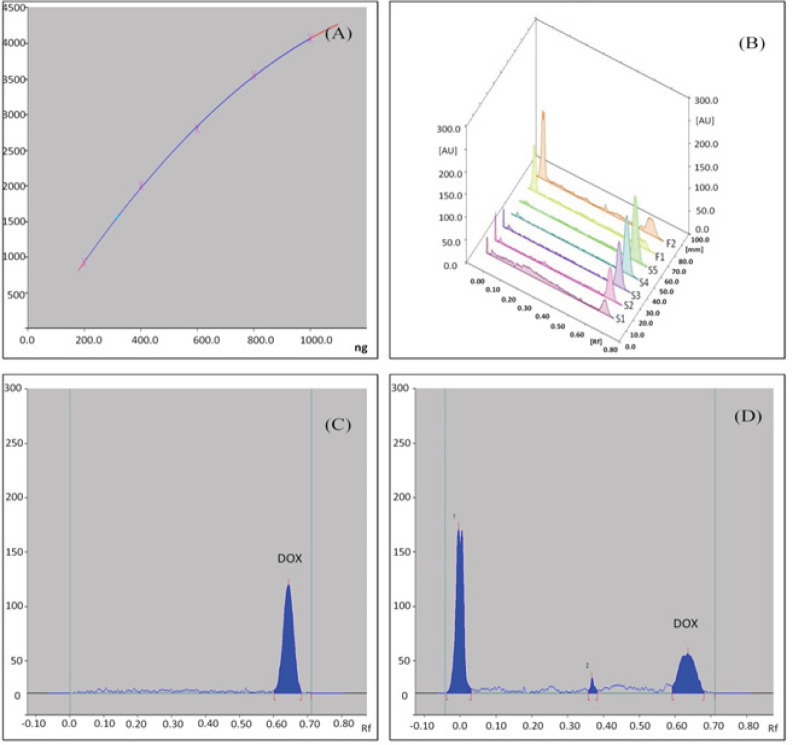
(A), Calibration curve for standard Dox; (B), Densitometric chromatogram of Dox at 254 nm (3D View), S1–S5 concentration 1, 2, 3, 5 ng/spot standard Dox, F1- AP-AgNPs and F2- Dox-*AP*-AgNPs; (C), Densitometric chromatogram of Dox after derivatization at 254 nm; (D), Densitometric chromatogram of Dox-*AP*-AgNPs after derivatization at 254 nm

HPTLC method was found to be simple, precise, accurate and convenient method for rapid screening of active constituents present in the plant extract and plant mediated biogenic nanoparticles. Determination and quantitation were performed by densitometric scanning at 254 nm in reflection/absorbance mode. Linearity range for *Andrographolide* was 1-5 mcg/spot with correlation coefficient R^2^=0.99183 (Figure 5A and B) in the concentration range 1-5 mcg/spot and for Dox was 1-5 ng/spot with correlation coefficient R^2^=0.99974 in the concentration range 1-5 ng/spot (Figure 6A and B). This method gave compact spots at R_f_ 0.18 (Figure 5C) corresponding to Standard *Andrographolide*, *AP* extract (Figure 5D), *AP*-AgNPs (Figure 5E) and Dox-*AP*-AgNPs (Figure 5F)and spots at R_f_ 0.64 corresponding to standard Dox (Figure 6C) and Dox-*AP*-AgNPs (Figure 6D). HPTLC quantitative estimation of *Andrographolide* in the extract (A.P Extract), biogenetically synthesized silver nanoparticle and the ternary conjugate of Andropgrapholide, silver and Dox (Dox-*AP*-AgNPs) were found to be, 2.228±0.0004 µg/10µL, 0.7896±0.0005 mcg/10mcL and 0.7466±0.0005 mcg/10µL, respectively. In a similar manner, HPTLC quantitative estimation of Dox was determined for the ternary conjugate (Dox-*AP*-AgNPs) was found to be, 0.3172±0.0002 mcg/10µL (Table 3).

*Antioxidant Assay*

*DPPH free radical scavenging assay*

**Figure 7:**
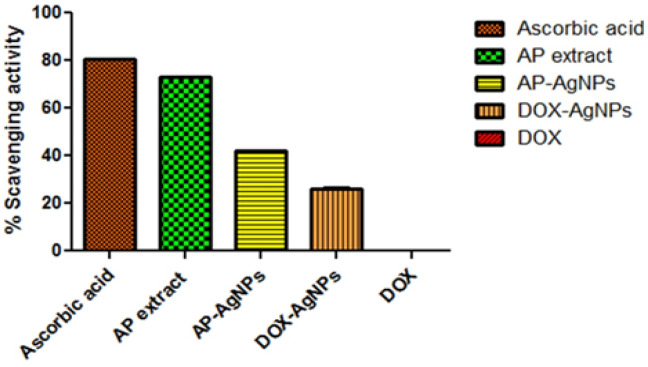
% Scavenging Activity of Formulations and Standard

The results displayed in Figure 7 indicate that the ascorbic acid and *AP* extract having maximum inhibition with a value of 80.4±0.026 and 72.9±0.077 %, respectively while no response was observed for the anticancer drug Dox alone. The *AP*-AgNPs demonstrated an inhibition of 42±0.152 %. The lesser inhibition shown by *AP*-AgNPs in comparison to *AP* extract may be due to the reduced concentration of extract in nanoparticles formulation. Further the % inhibition for the formulation namely Dox-*AP*-AgNPs was observed to be 26±0.608 % addition of Dox did not increased inhibition. Thus, it can be concluded that the free radical quenching ability of the *AP*-AgNPs might be due to the presence of components of *AP* in the synthesized *AP*-AgNPs. Evidence for such an induction of ROS leading to apoptosis and its role in down-regulating cell cycle progression by *Andrographolide* has recently been reported (Banerjee A et.al., 2017).

*MTT assay*

**Figure 8:**
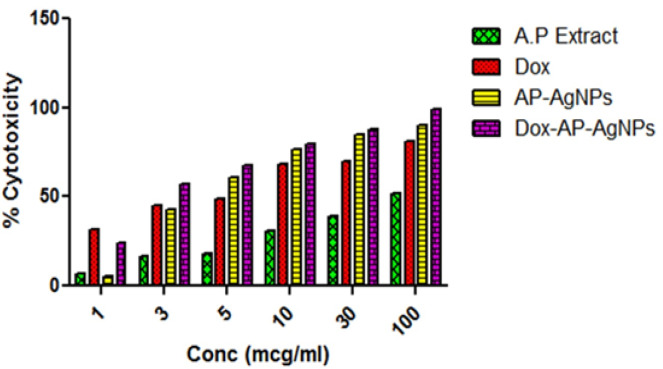
Percentage Cytotoxicity of AP Extract, *AP*-AgNPs and Dox-*AP*-AgNPs

MTT assay was carried out to investigate cytotoxic effect of freshly prepared nano formulations against MDA-MB-453, a triple negative breast cancer cell line. The percent inhibition of the MDA-MB-453, after 24 hours of post-treatment incubation is represented (Figure 8). The *AP*-AgNPs, exhibited greater percentage inhibition (90.38% at 100 mcg/mL) than, plant extract *AP* and standard Dox. The percentage inhibition of cell treated with the conjugated nano formulation (Silver+*AP*+Dox) indicated that Dox-AP-AgNPs showed higher percentage inhibition than AP-AgNPs (98.99% at 100 mcg/mL) in a dose dependent manner (p< 0.0001). Notably the Dox-AP-AgNPs showed 57.22 % at 3 mcg/mL itself while the AP-AgNPs, Dox and *AP* extract showed inhibition of 42.91, 45.38 and 16.66 % at 3 mcg/mL respectively. These values confirm that the ternary formulation containing the plant drug and Dox were responsible for the inhibition of MDA-MB-453 cell lines. The significant inhibition of Dox-*AP*-AgNPs indicates the synergistic effect of the ternary conjugate.

**Table 1:** Physicochemical Characterization of Biogenic Silver Nanoparticles

	DLS Analysis			Plasmon Resonance Peak (nm)	pH
Formulation Code	Particle Size (nm)	Zeta Potential (mV)	PDI		
*AP*-AgNPs	100±0.5	-32.26±0.4	0.586±0.0	417±0.5	7.3±0.1
Dox-*AP*-AgNPs	119±4.1	-19.8±0.2	0.637±0.0	437±1.0	4.3±0.5

**Table 2:** Stability Studies of Biogenic Silver Nanoparticles

Storage Conditions	Formulation	Duration (Month)	Particles Size (nm)	PDI	Zeta Potential (mV)	Plasmon Resonance Peak (nm)
*AP-*AgNPs	Room Temperature (23^o^C)	0	100±0.5	0.586±0.0	-32.2±0.4	417±0.5
		3	144±0.7	0.479±0.1	-22.6±0.2	417±0.3
		6	176±0.5	0.171±0.1	-17.2±0.2	419±1.5
	Refrigerator Temperature (4^o^C)	3	103±0.4	0.423±0.0	-30.5±0.1	417±0.5
		6	163±2.1	0.432±0.5	-28.2±0.4	418±1.0
Dox-*AP*-AgNPs	Room Temperature (23^o^C)	0	119±4.1	0.637±0.0	-19.8±0.2	437±1.0
		3	358±0.1	0.013±0.2	-14.5±0.3	437±0.7
		6	365±0.8	0.312±0.8	-13.7±0.3	438±1.3
	Refrigerator Temperature (4^o^C)	3	202±0.5	0.271±0.1	-19.2±0.2	437±0.4
		6	256±0.3	0.186±0.4	-16.9±0.1	437±0.9

**Table 3:** HPTLC Quantification of the Amount of *Andrographolide* and Dox Found in Biogenic Nanoparticles in mcg/µL

Formulation Code	Loading Sample (mcL)	*Andrographolide* (mcg) (Mean±SD) (n=3)	Dox (mcg) (Mean±SD) (n=3)
*AP* extract	10	2.228±0.0004	-
*AP*-AgNPs	10	0.7896±0.0005	-
Dox-*AP*-AgNPs	10	0.7466±0.0005	0.3172±0.0002

## Discussion

The study clearly showed that the Dox-*AP*-AgNPs conjugated forms are better in arresting proliferation in MDA-MB-453 breast cancer cell line and could provide anticancer effect compared to the standard Dox and *AP*-AgNPs alone. Dox-*AP*-AgNPs showed excellent cell inhibition rate due to their smaller size and spherical morphology. Participation of unsaturated lactone present in *AP* could have played a role in stabilization and synergy by sensitization of cancer cells as reported in literature (Zhou et al., 2008, Zhou et al., 2010). The higher rate of cancer cell inhibition of the ternary combination involving *AP* could be arising through nuclear condensation derived induction of apoptosis and cell cycle arrest observed in several cancers (Venkatadri et al., 2016, Babykutty et al., 2008; Forestier-Román et al., 2019). The cytotoxicity result shows that ternary formulation has greater advantage when it comes to inhibiting cancer cell line. Thus Dox-*AP*-AgNPs has higher level of inhibition towards MDA-MB-453 breast cancer cell line in a dose dependent manner when compared to *AP* extract, Dox and *AP*-AgNPs alone. 

Generally, stability and properties of *AP*-AgNPs synthesized by adopting green chemistry protocol has not been explored much. Stability of biogenically reduced *AP*-AgNPs was analyzed by measuring their particle size, zeta potential, PDI and plasmon resonance absorbance. We found slight changes in particle size, PDI and zeta potential of both *AP*-AgNPs and Dox-*AP*-AgNPs at room temperature but the values of these parameters of the formulations remained stable at 4.0±2.0°C (Table 2). However, both the storage conditions did not result in any aggregation or colour changes of the formulation. Slight changes in physicochemical properties but no agglomeration of nanoparticles was found. This could have arisen because the plant constituents might not have the potential to completely control the stability when compared to chemical stabilizing agents (p< 0.0001) (Table 2). Storing AgNPs at refrigerator temperature in dark mode can protect them from light and oxidation. AgNPs are typically susceptible to oxidation and this may be the reason for slight physicochemical changes of AgNPs when stored at room temperature.

This is the first time a ternary combination of natural drug, silver and anticancer drug is used for the successful inhibition of breast cancer cell lines paving a way to newer approach for cancer treatment. The nanoformulation containing *AP* extract as one of the constituents showed greater inhibitory activity against cancer cell line. However the biogenically synthesized ternary nanoformulation has greater potential to inhibit cancer cell better than raw plant ingredient or the binary *AP*-AgNPs, presumably due to synergistic effect. Support for such a synergy for the inhibition of breast cancer growth and metastasis has been observed for liposomal co-delivery of Dox and *Andrographolide* (Kang et.al., 2018). 

HPTLC quantification of this sample showed 2.23 µg/mL of *Andrographolide* and 0.95 mcg/mL of Dox, indicating that a lower concentration the plant drug along with Dox is sufficient enough to inhibit the breast cancer cell lines. Higher concentrations of the formulation Dox-*AP*-AgNPs, may not be suitable as the projected values of inhibition (2.9 fold increase for 746 mcg/mL) for the maximum concentration of the ternary conjugate showed only 84.99% inhibition as against the projected 1906%. This is a clear indication that there is an optimum inhibition at around 2.5 mcg/mL of *Andrographolide* and 0.95 mcg/mL of Dox the form of a ternary silver nano conjugate. 

In conclusion, the ternary conjugate of Dox-*AP*-AgNPs was found to inhibit the breast cancer cell lines containing 2.23 mcg/mL of *Andrographolide* and 0.95 mcg/mL of Dox. The beneficial effect of ternary conjugates could be attributed to extra stabilization of the *AP*-AgNPs coupled with DNA interaction and manifestation of anticancer activity through cell cycle arrest, nuclear condensation and induction of apoptosis as revealed by its DPPH free radical quenching with 42±0.152 % inhibition. Further, in-vitro and in-vivo studies are required for better understanding of most effective conjugated form. 
